# Does floral phenotyping reflect species integrity and natural hybridization of endangered *Calanthe* species on an oceanic island?

**DOI:** 10.3389/fpls.2026.1694101

**Published:** 2026-02-25

**Authors:** Seongjun Kim, Jung Eun Hwang, Chang Woo Lee, Hyeong Bin Park, Hwan-Joon Park, Young-Joong Kim

**Affiliations:** Research Center for Endangered Species, National Institute of Ecology, Yeongyang, Gyeongbuk, Republic of Korea

**Keywords:** backcrossing, *Calanthe aristulifera*, genetic diversity, introgression, morphological divergence, plant phenomics, population genetics

## Abstract

**Introduction:**

Floral phenotyping provides invaluable information for conserving genetic diversity of endangered plants. Endangered orchid, *Calanthe aristulifera* was selected to assess the applicability of phenotyping for identifying genetic variability and species integrity, given its morphological variations and evidences of natural hybridization on an oceanic island.

**Methods:**

Phenotyping using flower color and size (two phenotypes for each species) were compared with molecular data from genotyping-by-sequencing across *C. aristulifera* and coexisting allied (*Calanthe sieboldii*) and hybrid (*Calanthe* × *kibanakirishima*) species to track their species integrity and patterns in hybridization.

**Results and discussion:**

Principal coordinates analysis and phylogenetic clustering showed no genetic difference between the two phenotypes of *C. aristulifera*, by which *C. aristulifera* populations featured higher species integrity and lower genetic diversity than the coexisting allied species despite the divergence in floral morphology. Inversely, floral morphological divergence corresponded to interspecific genetic variability and the level of hybridization within *C.* × *kibanakirishima*. Hybrid index analysis particularly reflected the asymmetrical backcrossing and introgression toward *C. sieboldi* instead of *C. aristulifera* as a result of the similarities in floral morphology between the hybrids and *C. sieboldii*. Variations in flower lip and spur sizes were related to such genetic variations. In terms of biodiversity conservation, overall findings exhibit that divergence in floral morphologies may not ensure well-preserved genetic diversity or reduced species integrity of the endangered orchid. Some species may require the exigent protection of genetic diversity from the risk of genetic bottleneck, regardless of their marked phenotypic divergence.

## 1 Introduction

Floral phenotyping has been a key approach to understand biological mechanisms in plant science and their applicability to agriculture and plant breeding (Pieruschka and Schurr, 2019). Traditionally, floral phenotyping was used in common garden experiments to address the influence of environmental changes on plant growth and physiology, given the plant-environment interaction (Pieruschka and Poorter, 2012). Such studies reflected the ecological aspect of floral morphology, which can control natural selection by pollinators and the associated development of reproduction strategies to elevate progeny fitness (Juillet and Scopece, 2010). Phenotyping studies are recently combined with genetic assays to provides the information of the evolutionary processes, such as natural hybridization, adaptation to the surrounding environments, and ecological speciation (BerSweden et al., 2021; Sandro et al., 2019; Weber et al., 2020).

Combining floral phenotyping with genotyping is important to address the relationship between genes and morphological traits (Pieruschka and Poorter, 2012). Recently, rapid innovation of genotyping-by-sequencing (GBS) technique widens the applicability of such combined approaches by unveiling the influential factors on morphological polymorphisms, the relationship between phenotypic and genetic distances, and phylogenetic markers to differentiate the species having similar morphological traits (Andriamihaja et al., 2020; Dewoody et al., 2015; Lai et al., 2024). Such combinations of floral phenotypic and genetic studies have aided to accurately delimit the target endangered plant species or habitats, which is critical for conserving biodiversity (Joffard et al., 2022).

Orchids are one of the most threatened plant taxa because of their intrinsic rarity and horticultural value. Many orchid species have limited habitat range, small population size, and dependency upon the specific mycorrhizal symbiosis and pollinators (Phillips et al., 2020; Wraith et al., 2020). Consequently, many wild orchids are vulnerable to land use change, climate change, and biodiversity loss around their pristine habitats (Fay, 2018; Sarasan et al., 2025). Illegal harvest and smuggling are also the major threats declining the sustainability of wild orchid populations due to their economic value. In this context, orchids are globally considered to be critical indicators of biodiversity and endangered species requiring conservation programs (Calevo et al., 2025).

The phenotyping and genotyping approaches have been collaborated in conservation ecology to delimit the target for endangered orchid protection (Ahrens et al., 2017). For example, Cozzolino et al. (2006) suggested that the Mediterranean hybrid zones between *Orchis mascula* (L.) L. and *Orchis pauciflora* Ten. should deserve an elevated conservation priority as the potential stage for evolutionary processes, by comparing floral phenotyping and fruit set rate with genetic changes by hybridization. Evans et al. (2023) also adopted the combination of floral phenotyping and genotyping approaches to track the gene flow among the *Platanthera* hybrid complex in New Jersey and Maryland, and demonstrated that several hybrid zone should receive increasing conservation efforts to reduce the eroding species boundaries. However, unknowns still remain because primary research efforts have been concentrated to the model orchid genera such as *Phalaenopsis* and *Dendrobium* (Zhang et al., 2022). Large intraspecific divergence may also embarrass the detailed researches and conservations for orchids, by confounding which morphological traits and molecular markers can reflect the interspecific differences to clearly distinguish the target orchid species from the allied species (Joffard et al., 2022; Lee et al., 2008).

*Calanthe* R. Br. is the largest genus comprising 207 species in the Collabieae tribe of the Orchidaceae family, inhabiting across Asia, Pacific Island, Africa, and Australia (Nanjala et al., 2022). *Calanthe* species generally feature creeping rhizomes and underground pseudobulb systems, with which asexual propagation occur in the wild as well as the artificial cultivation conditions. Many *Calanthe* species recently received increasing conservation efforts because of the elevating risk of extinction by unsustainable overexploitation for horticulture and pharmacy (Nanjala et al., 2022), and global climate change (Qiu et al., 2023). Several population dynamics studies also emphasized the necessity of habitat management for several deceptive species like *Calanthe aristulifera* Rchb. (Kim et al., 2026), *Calanthe reflexa* Maxim. (Sakata et al., 2014), *Calanthe amamiana* Fukuyama (Sugiura, 2022), and *Calanthe discolor* Lindl. (Suetsugu and Fukushima, 2014), given that they are experiencing severe reproductive constraints under pollinator scarcity. In addition, insular populations of *Calanthe hoshii* S. Kobay. suffered from genetic diversity loss by decreased population size and inbreeding, reflecting the importance of genotyping-based population monitoring and relevant breeding programs (Katafuchi et al., 2024).

**Figure 1:**
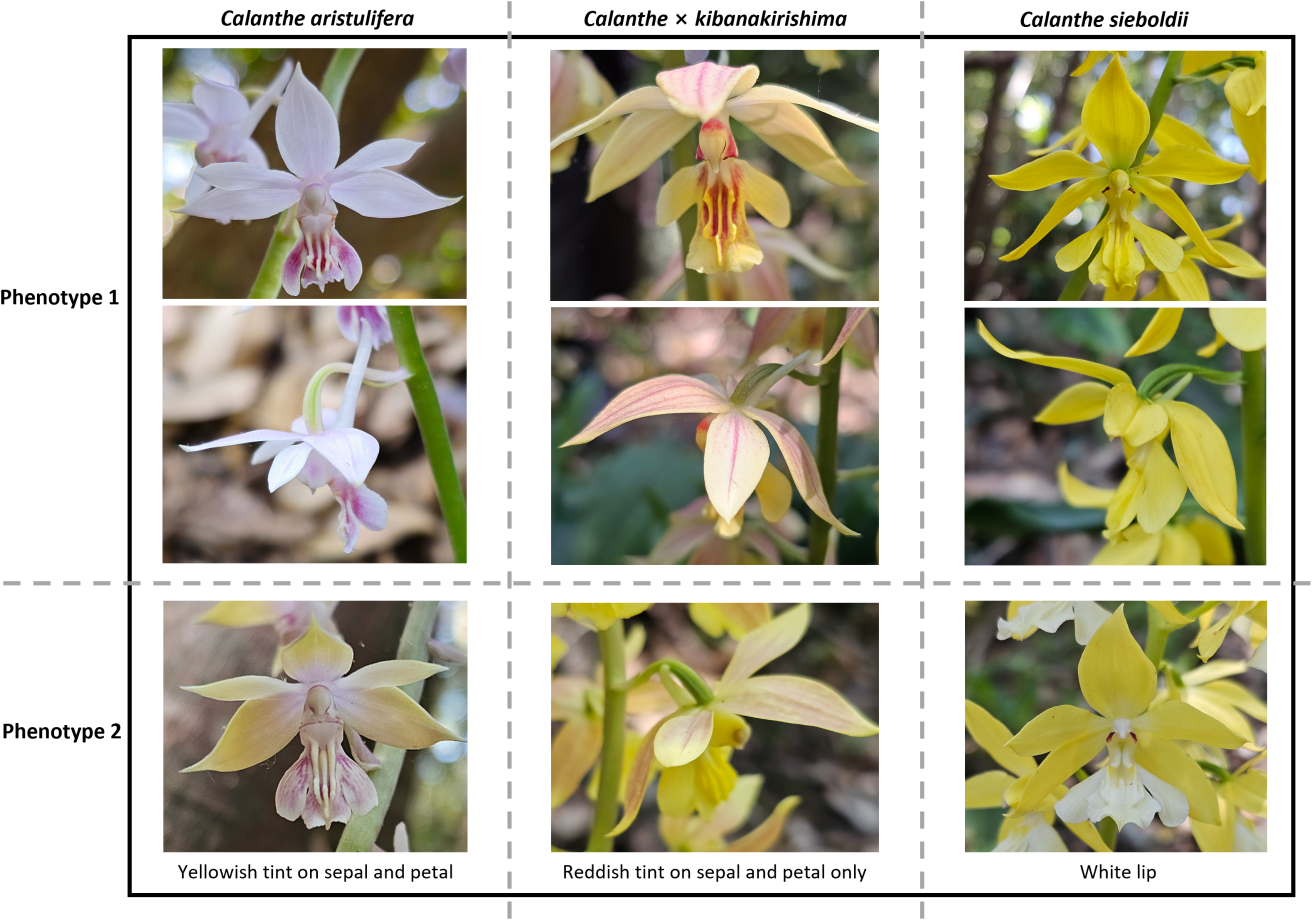
Flowers of each phenotype (phenotypes 1 and 2) of wild *Calanthe aristulifera*, *Calanthe* × *kibanakirishima*, and *Calanthe sieboldii*. Phenotype 1 individuals are dominant in wild populations compared with phenotype 2 individuals.

*C. aristulifera* is an evergreen orchid living in organic-rich warm-temperate evergreen forests in China, Japan, Taiwan, and South Korea (Hong et al., 2009; Li et al., 2021; Suetsugu et al., 2016). This species genetically close to several North East Asian species like *Calanthe sieboldii* Decne. ex Regel., *Calanthe bicolor* Lindl., and *C. discolor* Lindl. (Peng et al., 2024; Srikanth et al., 2013). From the perspective of morphology, the target species generally features the white to pale purple flower color, long ascending spur, and smaller size of sepal, petal, and lip relative to the allied *Calanthe* species (**Figure 1**). This species has the food-deceptive pollination mechanism without autonomous self-pollination requiring specific bee species like *Eucera nipponensis* and *Lasioglossum occidens* (Suetsugu et al., 2016; Suhara, 1993). Because of severe poaching*, C. aristulifera* is one of the legally protected orchid species as the endangered species grade II by the Ministry of Environment of South Korea and as CITES appendix II globally.

South Korean populations of *C. aristulifera* coexist with an allied species *C. sieboldii*, with which natural hybrid (*Calanthe* × *kibanakirishima* F. Maek., **Figure 1**) has been reported from Hongdo Natural Reserve in South Korean (Hong et al., 2010). This natural hybrid generally has the purple to red tint on the pale yellow flowers, of which size is intermediate between *C. aristulifera* and *C. sieboldii* (phenotype 1 of *C.* × *kibanakirishima* in **Figure 1**). Furthermore, possible phenotypic intermediates between the parent species and hybrids have also been observed from Hongdo Natural Reserve, such as *C. aristulifera* with yellowish tints on sepal and petal (phenotype 2 of *C. aristulifera* in **Figure 1**) and *C.* × *kibanakirishima* having a pure yellow lip without reddish tint (phenotype 2 of *C.* × *kibanakirishima* in **Figure 1**). Nonetheless, whether these phenotypic variations result from repetitive hybridization and introgression among those species still remained unclarified because of the lack of population genetics study. Therefore, combining floral phenotyping with genotyping is needed to identify species integrity and genetic diversity of *C. aristulifera* so that future restoration programs can delimit conservation priorities and strategies.

Our study aimed to confirm that variation in floral morphology can reflect genetic diversity and species integrity in threatened *Calanthe* species. Our primary null hypothesis was that some phenotypic patterns like the yellowish tint of *C. aristulifera* flowers may result from the hybridization with coexisting allied species featuring yellow flowers (*C. sieboldii*), and thus *C. aristulifera* individuals with the yellowish tint can be distinguishable from the individuals without such patterns in terms of population genetics. To answer this research hypothesis, phenotyping with floral morphology and GBS were applied to the wild populations of the target species and coexisting allied species on an oceanic island. Considering the lack of a reference nuclear genome for *C. aristulifera*, PacBio sequencing was used to acquire a *de novo* draft genome of *C. aristulifera* to detect genetic variations across *C. aristulifera* and its allied species using SNPs.

## 2 Methods

### 2.1 Study site and field sampling

**Figure 2:**
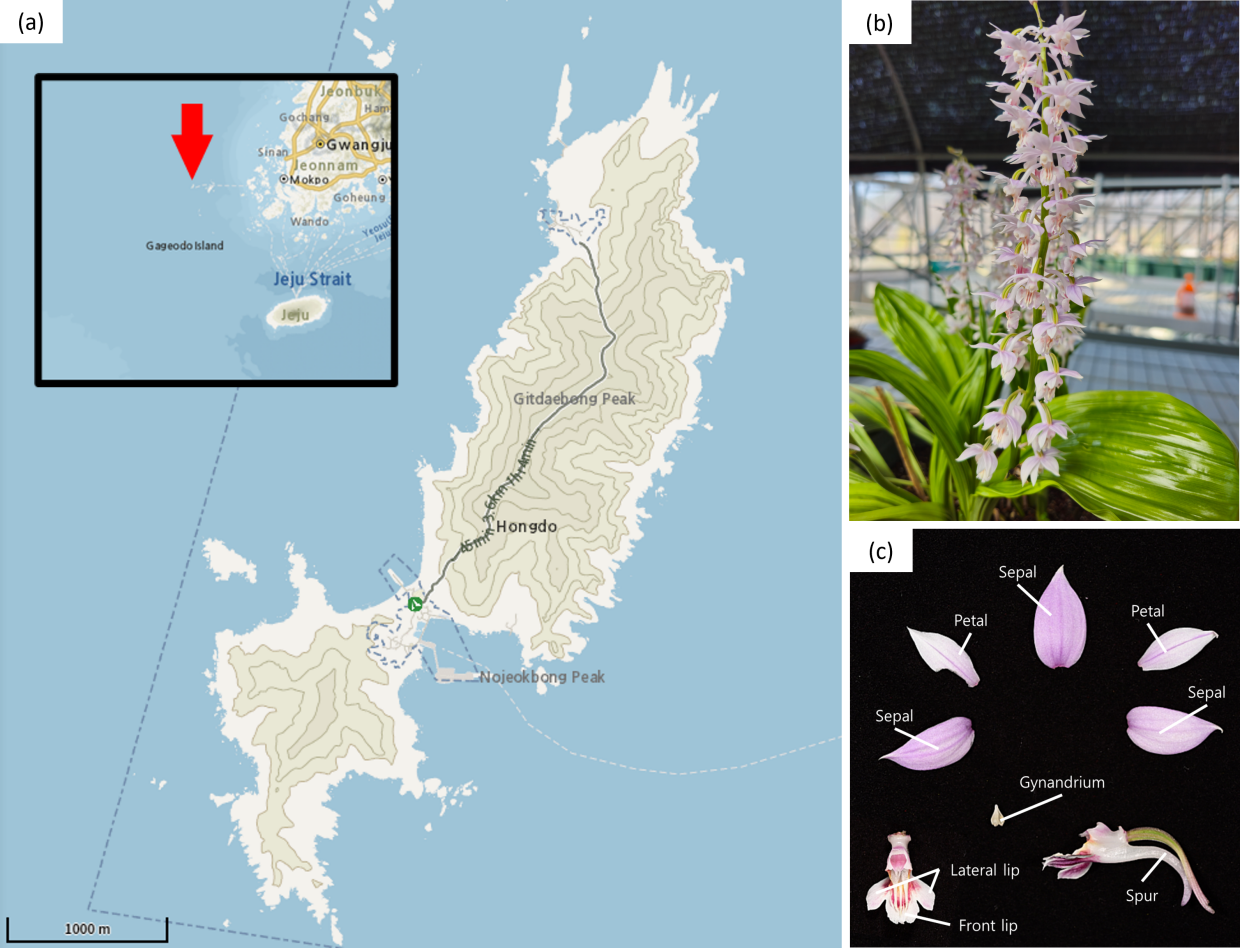
Location of study site **(a)**, the *Calanthe aristulifera* individual used for the draft genome assembly **(b)**, and anatomy of *C. aristulifera* flower **(c)**.

Study site was the largest Korean natural habitat of *C. aristulifera* in the Hongdo Natural Reserve (125˚12`E, 34˚41`N) (**Figure 2**). The study site is an oceanic island 110 km apart from the Korean Peninsula, and well protected as Dadohaehaesang National Park since 1981. A total of 253 C*. aristulifera* individuals were found from 12 populations within approximately 1.5 km^2^ area of the study site in May 2024 (phenotype 1: 225 individuals, phenotype 2: 28 individuals, **Figure 1**). These 12 populations contained 1–70 *C. aristulifera* individuals within 4–16 m^2^ microhabitat area, and were spatially separated each other (average distance between populations: 205 m). The *C. aristulifera* populations had the similar habitat environments, including warm-temperate climate, organic-rich acidic soils, and low altitude range (190–330m). Near *C. aristulifera* populations, 50 C*. sieboldii* (phenotype 1: 48 individuals, phenotype 2: 2 individuals, **Figure 1**) and 29 C. × *kibanakirishima* (phenotype 1: 26 individuals, phenotype 2: 3 individuals, **Figure 1**) individuals coexisted.

In May 2024, 1–6 *C. aristulifera* individuals were randomly sampled from each of the 12 C*. aristulifera* populations for analyzing SNPs (n = 36 for *C. aristulifera*), given the varying number of individuals per populations. Size of sampling plot for each population (12 in total) was 4 × 4 m to account for varying microhabitat area (4–16 m^2^). To minimize unintended clonal sampling, only one sample was collected from the ramets from a single rhizome system (minimum distance between sampled individuals: 50 cm). Other coexisting *C. sieboldii* (n = 16) and *C*. × *kibanakirishima* (n = 12) within sampling plot were also. Total number of sampled *Calanthe* individuals was 64. In May 2024, fresh leaves of the selected *Calanthe* individuals were sampled, stored in an icebox (4 °C), and brought to the laboratory until SNP analysis.

### 2.2 Floral phenotyping

Floral morphological traits including the flower number per individual, length and width of sepal, petal, front lip, and lateral lip, length and diameter of spur, and length of gynandrium were measured simultaneously with the leaf sampling in May 2024 for each sampled individual (**Figure 2c**). Three healthy flowers per individual were measured, of which values were averaged for each individual. This morphological measurement was conducted using a digital caliper (error range: ± 0.02 mm) (CD-20APX, Mitutoyo, Japan) by a single person to avoid the unintended measurement inconsistency.

Each sampled *Calanthe* species was subdivided into the two phenotypes in accordance with the color of the fresh flowers (**Figure 1**). For example, the phenotype 1 of *C. aristulifera* (CA1) has the white to pale purple petal and sepal like the reference individual for the draft genome assembly (**Figure 2b**), whereas the phenotype 2 of *C. aristulifera* (CA2) has the additional yellowish tint on the petal and sepal. Unlike the phenotype 1 of *C.* × *kibanakirishima* (CK1), the phenotype 2 of *C.* × *kibanakirishima* (CK2) lacks the reddish tint on the lip. While the flower of the phenotype 1 of *C. sieboldii* (CS1) is characterized by a bright yellow color, the lip color of the phenotype 2 of *C. sieboldii* (CS2) is white. Consequently, the number of sampled individuals for each phenotype was 30 for AP1, 6 for AP2, 9 for KP1, 3 for KP2, 14 for SP1, and 2 for SP2, respectively.

### 2.3 Draft genome

As no reference nuclear genome was available for the target species, a draft genome assembly was conducted as well. Leaf samples for draft genome were collected in April 2024, from a reference *C. aristulifera* individual growing in a greenhouse of Research Center for Endangered Species of South Korea (129˚09`E, 36˚38`N) (**Figure 2b**). This reference individual was harvested for *ex-situ* conservation and artificial propagation experiment purposes in 2020 from the wild habitat where the first Korean voucher specimen of *C. aristulifera* (voucher code: G.H. Jang et al. 035401, CNU) was recorded at Hongdo Natural Reserve (Hong et al., 2009).

Genomic DNA of the leaf samples was extracted with the Aprep Total DNA KIT (APBIO, Namyangju, South Korea) following the manufacturer’s instructions. Then, the extracted DNA was quantified using a Thermo Scientific Nanodrop 8000 spectrophotometer (Fisher Scientific, Waltham, MA, USA), and sequenced with one Revio SMRT cell tray of PacBio Revio (Pacific Biosciences of California, Inc., Menlo Park, CA, USA). According to the PacBio Sample Net-Shared Protocol (https://www.pacb.com/), short, low-quality reads and adaptor sequences were discarded from the obtained raw reads. Basecalling was performed with the onboard analysis software embedded in the PacBio Revio system. Whole genome sequencing library of the PacBio HiFi long reads was constructed with QIAseq FX DNA Library CDI Kit (Qiagen, Hilden, Germany). A *de novo* draft genome was assembled using HiFiasm v.0.19.9-r606 (Cheng et al., 2021). The haplotype-resolved genome assembly was conducted at the HiFi-only assembly mode (*k*-mer length = 51, minimizer window size = 51, error correction rounds = 3, assembly cleaning rounds = 4, bubble popping threshold = 10 Mb).

### 2.4 SNP identification and filtration

Genomic DNA for SNP analysis was extracted from the 64 samples with the Aprep Total DNA KIT (APBIO, Namyangju, South Korea) and quantified using a Thermo Scientific Nanodrop 8000 spectrophotometer (Fisher Scientific, Waltham, MA, USA). The extracted DNA was digested with the ApeKI enzyme (GCWGC). Size of GBS library fragments was 300–600 bp with a major peak at 480–500 bp, which was reorganized into short reads (2 PE × 151 bp) according to Elshire et al. (2011); Oh et al. (2023) by paired-end sequencing using the Illumina NovaSeq X platform (Illumina Inc., CA, USA). Low-quality short reads were trimmed using Trimmomatic v.0.39 (window size: 4, leading and trailing: ≧ 3, mean quality: ≧ 15, read length: ≧ 36 bp) (Bolger et al., 2014). Then, BWA-MEM2 was used to mapped the filtered Illumina short reads to the draft genome of *C. aristulifera* with (Li, 2013). Raw SNPs were identified with DeepVariant v.1.6.0. software (model type: Illumina WGS) (Yun et al., 2021), and firstly filtered at the criteria of minimum depth ≥ 3, minor allele frequency > 5%, missing data < 30%. Finally, only biallelic SNP loci with no missing values throughout all 64 samples were used for the statistical analyses (a total of 3382 SNPs).

### 2.5 Statistical analyses

Two-way analysis of variance (ANOVA) was used to test the effects of species and phenotype on the floral morphological traits, with the *post-hoc* pairwise comparisons using Scheffe test (α = 0.05) using the agricolae (De Mendiburu and Simon, 2015) of R v.4.3.2. software (R Core Team, 2023). All floral morphological trait data were log-transformed before statistical analyses for normalization. In addition, the primary genetic diversity indices including expected heterozygosity (He), observed heterozygosity (Ho), Nei’s genetic diversity (GD), polymorphism informative content (PIC), and minor allele frequency (MAF) were calculated to quantify the patterns in heterozygosity and polymorphism using the snpReady (Granto et al., 2018) packages of R v.4.3.2. software (R Core Team, 2023).

Principal coordinates analysis (PCoA) was used to exhibit the multivariate patterns in the floral morphology and genetic variability. Floral morphological data for PCoA were standardized by Z-transformation. Permutational multivariate analysis of variance (PERMANOVA) and permutational analysis of multivariate dispersion (PERMDISP) with 9999 permutations were then conducted to evaluate the effects of species and phenotype on the multivariate group centroids and dispersions of floral morphological traits and genetic data (α = 0.05). In PCoA, PERMANOVA, and PERMDISP, Bray-Curtis dissimilarity and Jaccard index were used as this distance matrix for floral morphological and genetic data, respectively. These analyses were performed using the vegan (Oksanen et al., 2024) and ecodist (Goslee and Urban, 2007) packages of R v.4.3.2. software (R Core Team, 2023).

STRUCTURE v.2.3.4. software was used for Bayesian clustering to infer genetic admixture proportions among the 64 samples (burn in: 10000, Markov chain Monte Carlo simulation: 100000, iteration: 10), for which the maximum likelihood (L(k)) and Δk methods were applied to select the number of clusters (k value) for visualization (Pritchard et al., 2000). Based on the number of clusters from the maximum likelihood (L(k)) and Δk methods, neighbor-joining phylogenetic tree with Euclidean distance and heatmap analyses were implemented to describe the genetic relationship among the phenotypes of *C. aristulifera*, *C. sieboldii*, and *C*. × *kibanakirishima* using the factoextra (Kassambara and Mundt, 2016) and ComplexHeatmap (Gu et al., 2016) packages of R v.4.3.2. software (R Core Team, 2023). As no species-specific marker was currently available to distinguish the three studied *Calanthe* species, the 3382 SNPs were provisionally clustered on the basis of the average silhouette width method (k = 10) for the heatmap analysis, given the lack of such grouping procedure for SNPs in the STRUCTURE analysis. This approach is adopted to seek any diagnostic (species-specific) SNPs to distinguish the two parent species (Hamilton et al., 2013), considering that the absence of diagnostic markers induces overestimation of interspecific heterozygosity for the parent species individuals.

Using the detected diagnostic SNPs, we estimated hybrid index and interspecific heterozygosity for each combination of species and phenotype with the introgress package (Gompert and Buerkle, 2010) in R v.4.3.2. software. Here, hybrid index indicates the proportion of alleles inherited from one of the parent taxa. Based on theoretical expectation, this analysis assumed that pure *C. sieboldii*, pure *C. aristulifera*, and their F1 hybrid should have a hybrid index of 0, 1, and 0.5, respectively (Milne and Abbott, 2008), and multiple generations of hybridization and genetic backcrosses should decrease interspecific heterozygosity (Suetsugu et al., 2024). Accordingly, individuals with low (< 0.25) or high (> 0.75) hybrid index are considered to experience genetic backcrossing for multiple generations, while individuals having intermediate hybrid index (0.25–0.75) with a high interspecific heterozygosity (near 1) represent recent hybrid generations (F1 and F2) (Milne and Abbott, 2008).

Multiple matrix regression with randomization (MMRR) with 9999 permutations was conducted to examine the linear relationship between morphological and genetic distances using the ecodist package (Goslee and Urban, 2007) of R v.4.3.2. software (R Core Team, 2023). Here, Bray-Curtis dissimilarity and Jaccard index were adopted as a matrix of morphological and genetic distances, respectively. Canonical analysis of principal coordinates (CAP) with 9999 permutations was applied to assess the independent, marginal effect of each floral morphological trait on genetic variability using the vegan (Oksanen et al., 2024) package of R v.4.3.2. software (R Core Team, 2023).

## 3 Results

### 3.1 Floral morphological traits

**Figure 3:**
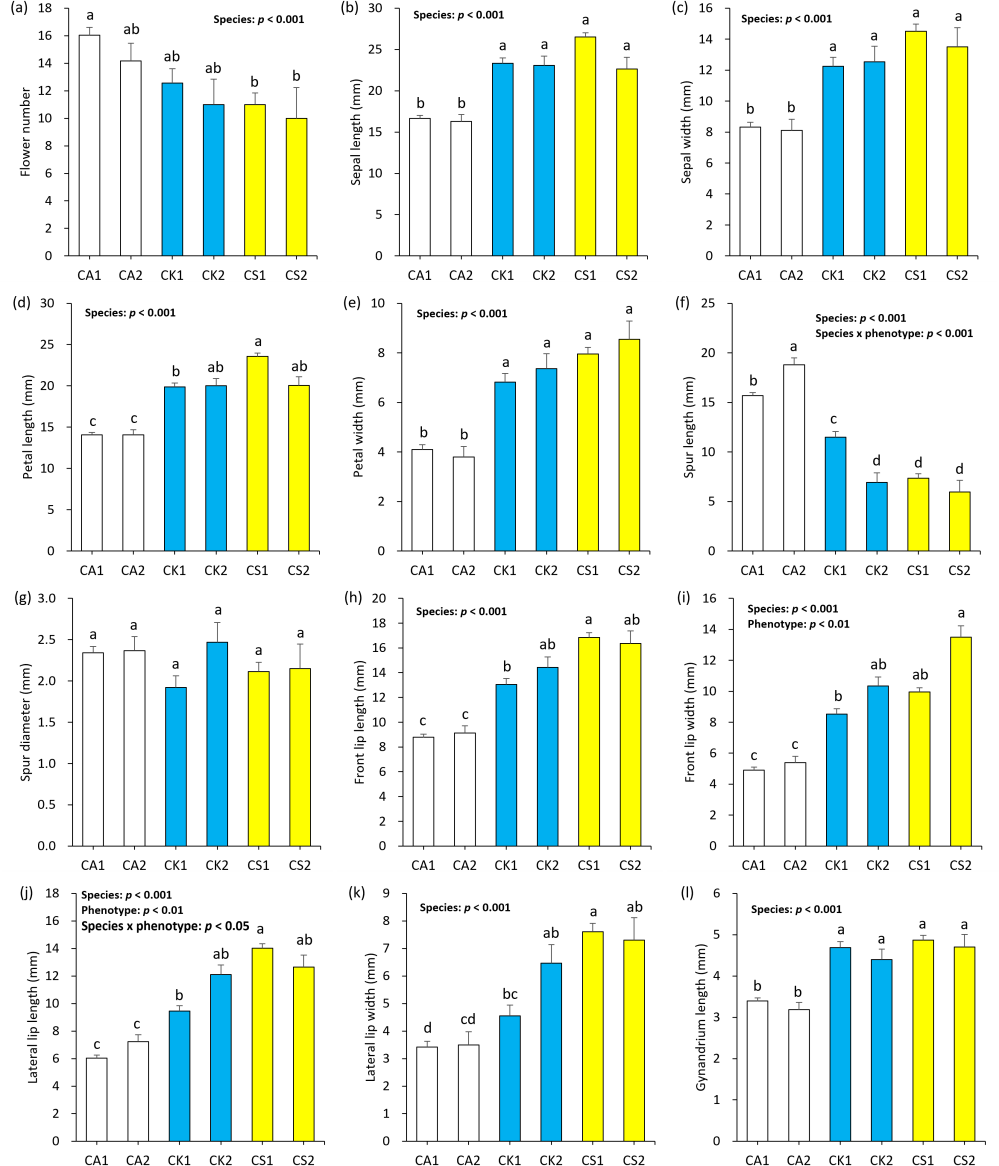
Difference in floral morphology according to species (*Calanthe aristulifera*, CA, *Calanthe* × *kibanakirichima*; CK, *Calanthe sieboldii*; CS) and phenotype (1 and 2). Color of each bar graph represents species (white: CA, blue: CK, yellow: CS). Letters above error bars indicate the statistical significance by the *post-hoc* Scheffe test at *p* < 0.05. Only the explanatory variables significant at *p* < 0.05 in Two-way ANOVA are presented on each panel. Each panel includes the results on flower number **(a)**, sepal length **(b)**, sepal width **(c)**, petal length **(d)**, petal width **(e)**, spur length **(f)**, spur diameter **(g)**, front lip length **(h)**, front lip width **(i)**, lateral lip length **(j)**, lateral lip width **(k)**, and gynandrium length **(l)**.

Two-way ANOVA showed the significant effects of species on the floral morphological traits except for spur diameter (*p* < 0.001, **Figure 3**). Phenotype and species × phenotype interaction had the significant effects on spur length (*p* < 0.001, **Figure 3f**), front lip width (*p* < 0.01, **Figure 3i**), and lateral lip length (*p* < 0.05, **Figure 3j**) only. *Post hoc* Scheffe test showed that CA1 and CA2 generally exhibited the higher flower number (**Figure 3a**), longer spur length (**Figure 3f**), and smaller sepal (**Figures 3b, c**), petal (**Figures 3d, e**), front lip (**Figures 3h, i**), lateral lip (**Figures 3j, k**), and gynandrium (**Figure 3l**) than CK1, CK2, CS1, and CS2. However, differences in floral morphological traits between the phenotypes within each species were not significant, except for spur length of *C. aristulifera* and *C.* × *kibanakirishima* (**Figure 3f**). CK1 was the intermediate between CA1 and CS1 in terms of petal length (**Figure 3d**), petal width (**Figure 3e**), spur length (**Figure 3f**), front lip length (**Figure 3h**), lateral lip length (**Figure 3j**), and lateral lip width (**Figure 3k**). Floral morphological traits of CK2 did not significantly differ from CS1 (**Figure 3**).

### 3.2 Draft genome and filtered SNPs

Pacbio sequencing established 5.1 million HiFi long reads with 84.39 Gbp length in total, which were used for the *de novo* draft genome assembly. Average read length and N50 for the HiFi long reads were 16,546 bp and 16,985 bp. HiFi sequencing coverage was approximately 7.6×. The obtained reference draft genome comprised 3.6 thousand scaffolds with 9.4 Gbp length in total, each of which had average length and N50 values of 0.26 Gbp and 0.49 Gbp.

278.8 Gbp of Illumina short reads were acquired from the 64 sample (GC value: 47%, Q30 value: 94%). Average number and total length of raw reads per sample were 24,802,946 and 3.6 Gbp. Total length of trimmed and mapped read per sample was 11.3 Mbp, and average coverage depth per SNP was 93.97×. Accordingly, a total of 3382 filtered SNPs were detected and used for analyzing genetic variations across *C. aristulifera*, *C.* × *kibanakirishima*, *C. sieboldii*. Genetic diversity indices including He, Ho, GD, PIC, and MAF based on the 3382 SNPs are shown in **Table 1**. CK1 and CA2 showed the highest and lowest He, Ho, GD, PIC, and MAF among the studied species and phenotypes (**Table 1**).

**Table 1:** Genetic diversity indices of each phenotype of *Calanthe aristulifera* (CA), *Calanthe* × *kibanakirishima* (CK), and *Calanthe sieboldii* (CS). Value for each phenotype (1 or 2) refers to the same phenotype with the same number in **Figure 1**.

Species	Phenotype	He^A^	Ho	GD	PIC	MAF
CA	1	0.13	0.12	0.13	0.11	0.09
	2	0.11	0.11	0.11	0.09	0.08
CK	1	0.21	0.32	0.21	0.17	0.17
	2	0.13	0.25	0.13	0.10	0.12
CS	1	0.20	0.19	0.20	0.16	0.15
	2	0.14	0.19	0.14	0.11	0.12

^A^, He, expected heterozygosity; Ho, observed heterozygosity; GD, Nei’s genetic diversity; PIC, polymorphism informative content; MAF, minor allele frequency.

### 3.3 Morphological and genetic variations

**Figure 4:**
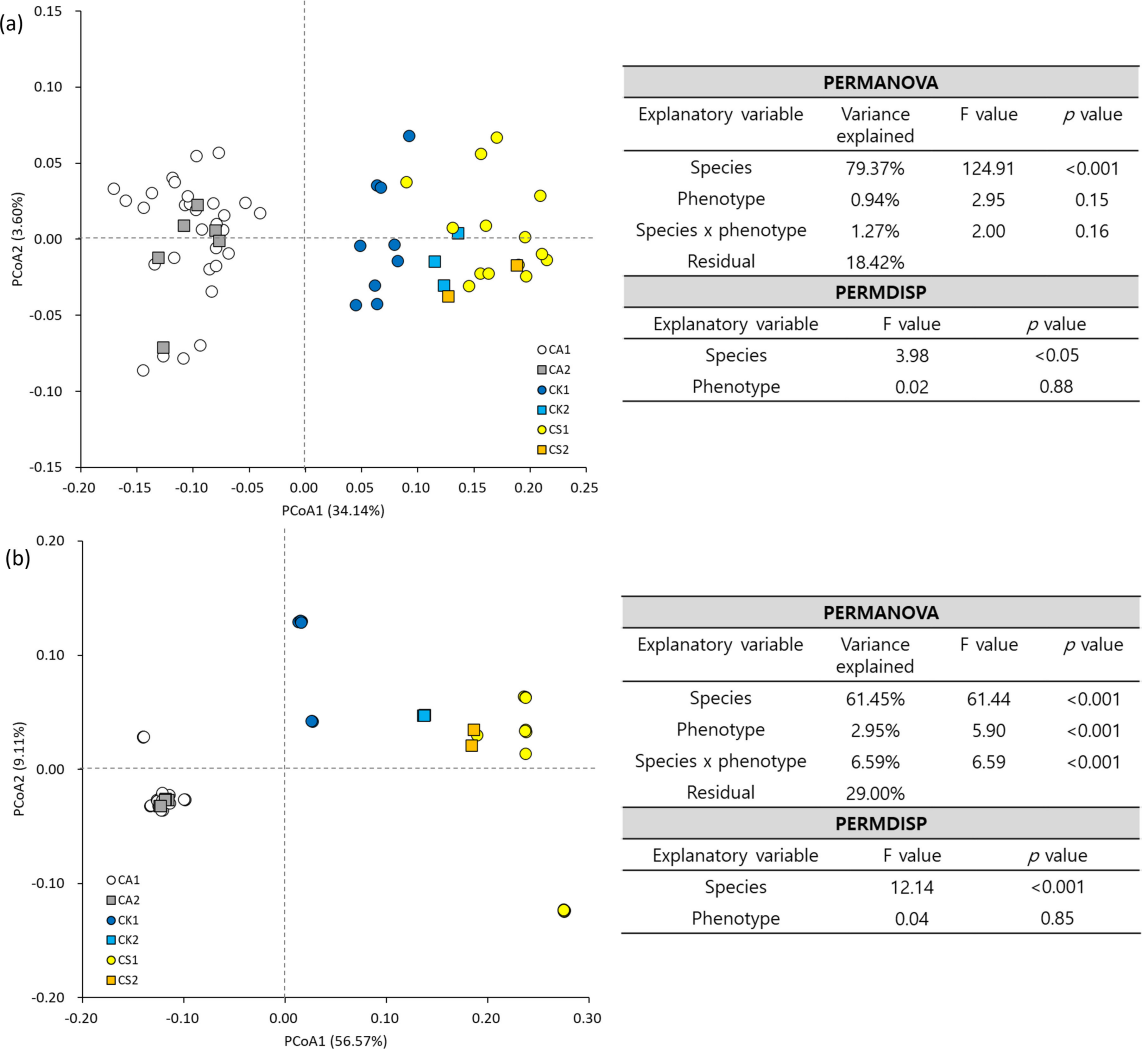
Principal coordinates analysis (PCoA), permutational multivariate analysis of variance (PERMANOVA), and permutational analysis of multivariate dispersion (PERMDISP) for floral morphology **(a)** and 3382 SNPs **(b)** (CA1: phenotype 1 of *Calanthe aristulifera*, CA2: phenotype 2 of *C. aristulifera*, CK1: phenotype 1 of *Calanthe* × *kibanakirishima*, CK2: phenotype 2 of *C*. × *kibanakirishima*, CS1: phenotype 1 of *Calanthe sieboldii*, CS2: phenotype 2 of *C. sieboldii*). Bray-Curtis dissimilarity and Jaccard index were used for floral morphological and genetic data, respectively.

The first three PCoA axes explained 34.14, 3.60, and 2.40% and 56.57, 9.11, and 8.16% of the morphological and genetic variations, respectively (**Figure 4**). In the PCoA, *C. aristulifera* was clearly distinguished from the other two species in terms of morphological (**Figure 4a**) and genetic variations (**Figure 4b**). CK1 was a genetic intermediate between *C. aristulifera* and *C. sieboldii* (**Figure 4b**) but slightly biased to *C. sieboldii* in terms of floral morphology (**Figure 4a**). CK2 was morphologically and genetically closer to *C. sieboldii* than *C. aristulifera* (**Figures 4a, b**). PERMANOVA and PERMDISP exhibited that species had the significant effects on multivariate centroids and dispersions of the morphological traits (**Figure 4a**) and 3382 SNPs (**Figure 4b**) (*p* < 0.001), whereas phenotype (*p* < 0.005) and species × phenotype interaction (*p* < 0.001) affected multivariate centroids of 3382 SNPs only (**Figure 4b**). Unlike *C.* × *kibanakirishima*, there was no clear genetic differentiation between the phenotypes of *C. aristulifera* and *C. sieboldii*, (**Figure 4b**). Individuals from a single population shared similar genetic patterns rather than floral morphology (**Figure 4**).

### 3.4 Phylogenetic clustering

**Figure 5:**
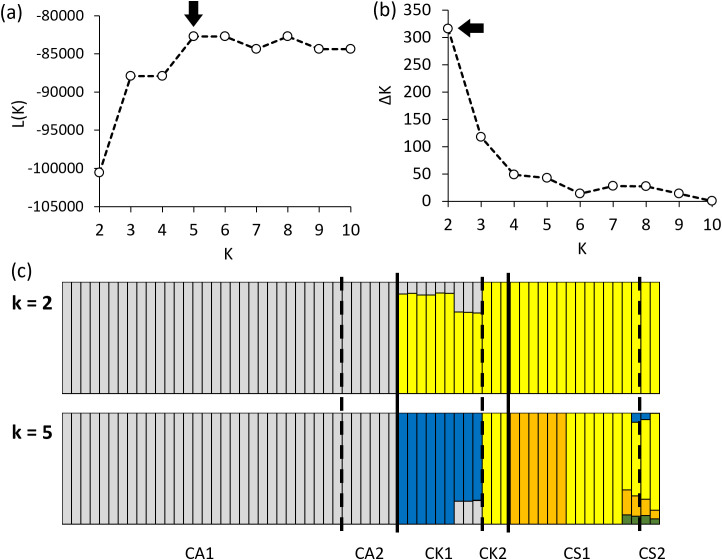
Selection of k value based on maximum likelihood **(a)** and delta k methods **(b)**, and STRUCTURE analysis results at 2 and 5 hypothetical ancestral groups **(c)** using 3382 SNPs (CA1: phenotype 1 of *Calanthe aristulifera*, CA2: phenotype 2 of *C. aristulifera*, CK1: phenotype 1 of *Calanthe* × *kibanakirishima*, CK2: phenotype 2 of *C*. × *kibanakirishima*, CS1: phenotype 1 of *Calanthe sieboldii*, CS2: phenotype 2 of *C. sieboldii*).

Clustering with maximum likelihood (L(K), **Figure 5a**) and ΔK (**Figure 5b**) suggested 5 and 2 as appropriate numbers of clusters for the 64 samples, respectively. The STRUCTURE analysis with k = 2 demonstrated that CA1 and CA2 were clearly distinguishable from the other species and CK2 was classified as the same group with CS1 and CS2 (**Figure 5c**). CK1 contained both hypothetical ancestral compositions from CA1and CS1, although it seemed to be biased to CS1 (**Figure 5c**). At k = 5, CK1 and several CS1 individuals were subdivided into the separate ancestral groups (**Figure 5c**).

**Figure 6:**
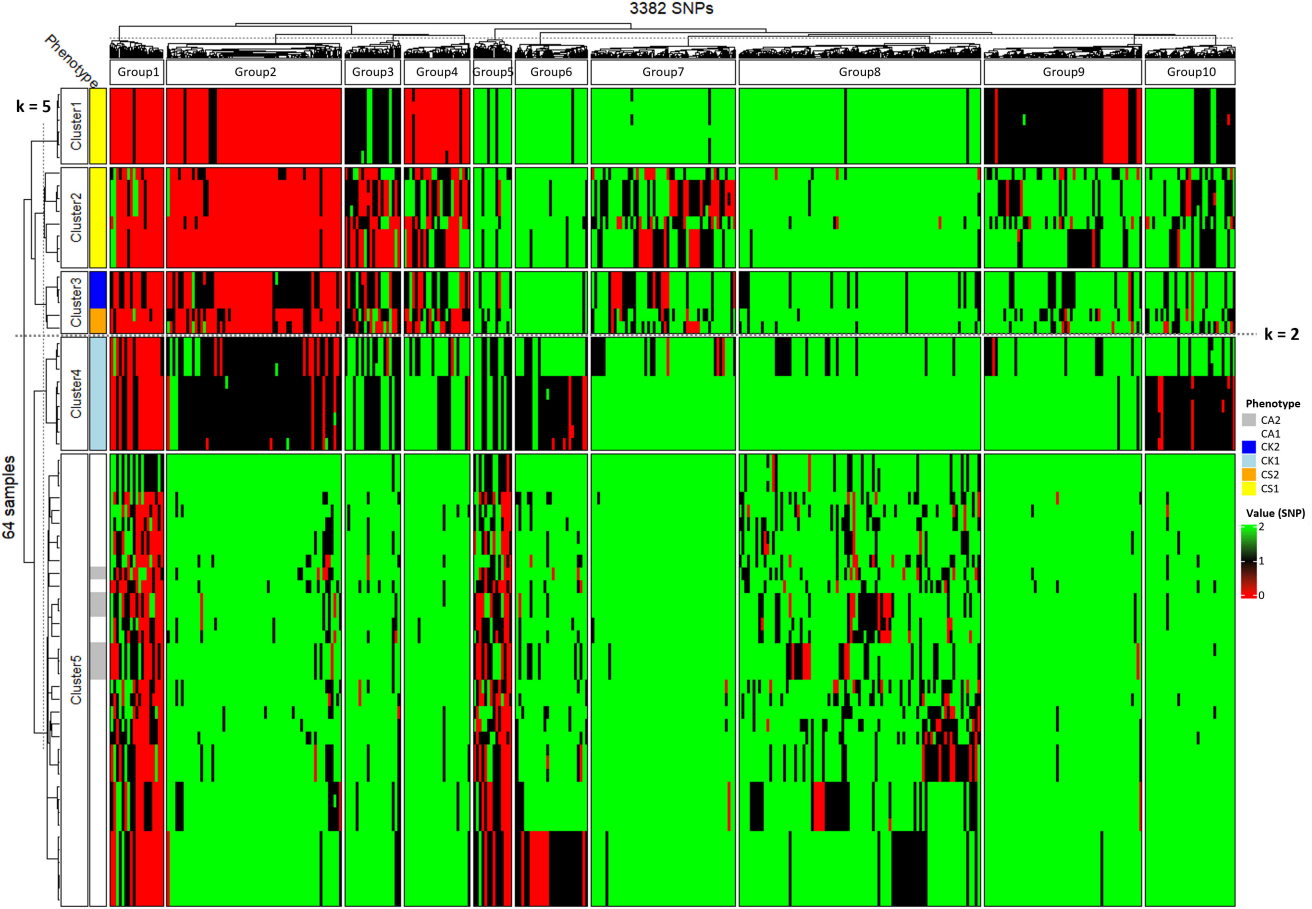
Heatmap and neighbor-joining clustering on 64 samples and 3382 SNPs (CA1: phenotype 1 of *Calanthe aristulifera*, CA2: phenotype 2 of *C. aristulifera*, CK1: phenotype 1 of *Calanthe* × *kibanakirishima*, CK2: phenotype 2 of *C.* × *kibanakirishima*, CS1: phenotype 1 of *Calanthe sieboldii*, CS2: phenotype 2 of *C. sieboldii*). K values are based on maximum likelihood (k = 5) and ΔK (k = 2) methods for the samples and average silhouette width (k = 10) for the SNPs, respectively. SNP value (0, 1, 2) indicates the number of same alleles with the *C. aristulifera* draft genome at each SNP.

Detailed phylogenetic clustering with a heatmap revealed that CA1 and CA2 were categorized into the single cluster at k = 2 and 5 (**Figure 6**). CK1 was separated from CA1 and CA2 at k =5, while CK2 was classified as the same cluster with CS2 at k = 5 (**Figure 6**). There were two CS1 clusters at k = 5 despite the similarity in the morphological traits. Meanwhile, only the SNP group2 (540 of 3382 SNPs) showed a clear genetic gradient across the three studied *Calanthe* species (**Figure 6**). For example, CA1 and CA2 were dominated by the homozygotes same with the reference *C. aristulifera* draft genome, while CS1 and CS2 were dominated by the homozygotes differing from the reference genome in the SNP group2 (**Figure 6**). CK1 was dominated by the heterozygotes in the SNP group2, which was the intermediate genetic pattern between *C. aristulifera* and *C. sieboldii* (**Figure 6**). CK2 featured the similar proportions of the heterozygotes and homozygotes differing from the reference genome in the SNP group2 (**Figure 6**), as the intermediate between CK1 and CS1. These patterns demonstrated that CK2 possibly resulted from hybridization and backcrossing between the earlier generation of hybrid (CK1) and the parent species (CS1).

### 3.5 Hybrid index

**Figure 7:**
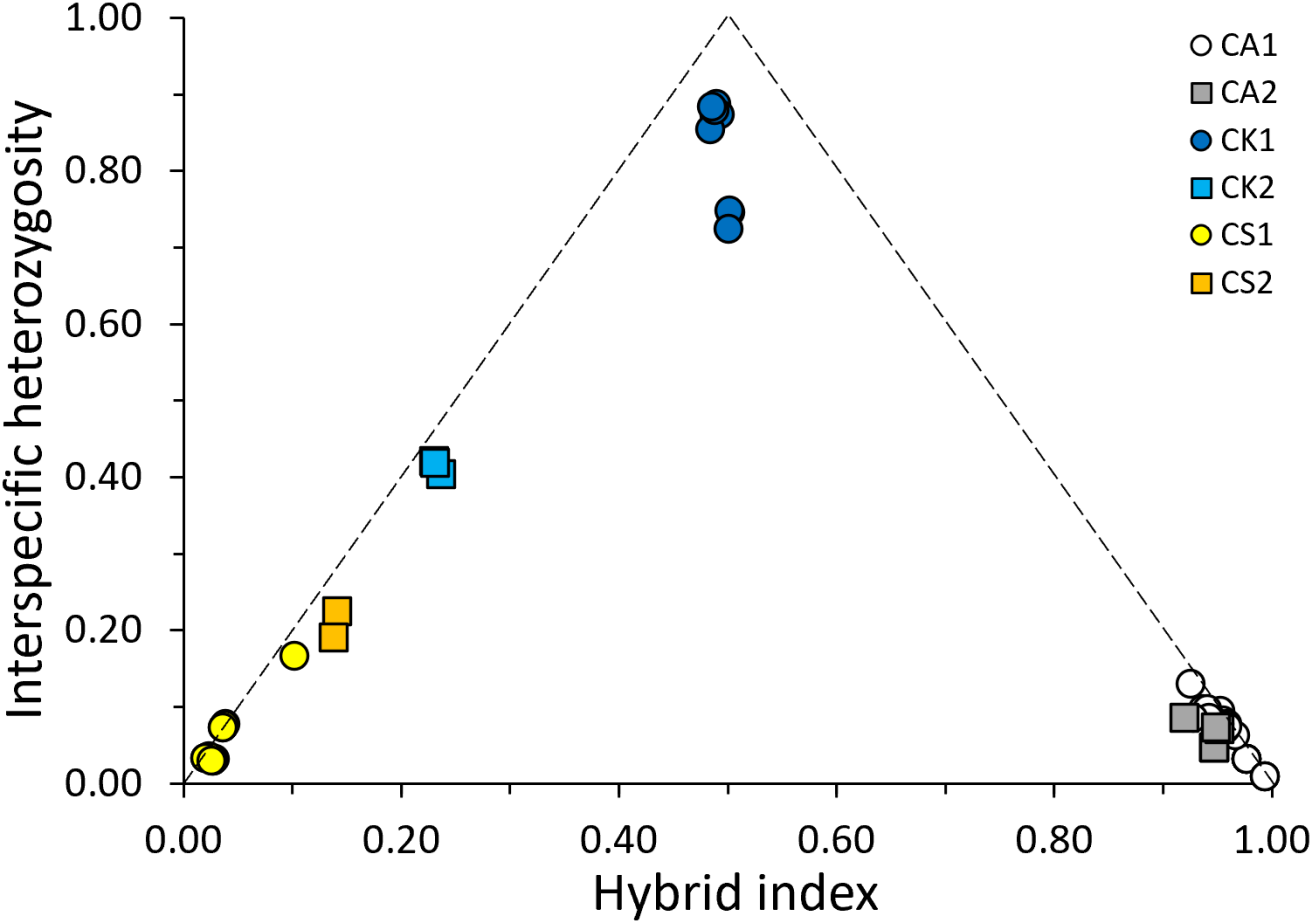
Triangle plot showing interspecific heterozygosity and hybrid index for the 64 sampled individuals using the species-specific 540 SNPs (CA1: phenotype 1 of *Calanthe aristulifera*, CA2: phenotype 2 of *C. aristulifera*, CK1: phenotype 1 of *Calanthe* × *kibanakirishima*, CK2: phenotype 2 of *C*. × *kibanakirishima*, CS1: phenotype 1 of *Calanthe sieboldii*, CS2: phenotype 2 of *C. sieboldii*).

Hybrid index and interspecific heterozygosity were estimated using a subset of 540 SNPs (the SNP group2 in **Figure 6**) as potential markers showing the species-specific genetic differences. Other SNP groups were excluded for hybrid index estimation because they tended to reflect the intraspecies genetic variations or did not differ between the two parent taxa (**Figure 6**). This analysis showed that CK1 and CK2 had a hybrid index of 0.48–0.50 and 0.23–24 (**Figure 7**). Hybrid index of CA1, CA2, CS1, and CS2 were 0.93–0.99, 0.92–0.95, 0.02–0.10, and 0.13–0.14, respectively (**Figure 7**). Meanwhile, interspecific heterozygosity of CK1 and CK2 were 0.72–0.89 and 0.40–0.42, which were generally higher than those of CA1 (0.01–0.13), CA2 (0.05–0.09), CS1 (0.03–0.17), and CS2 (0.19–0.22) (**Figure 7**). Lowered hybrid index and interspecific heterozygosity of CK2 than CK1 showed that multiple generations of hybridization and backcrossing possibly occurred toward *C. sieboldii*. Conversely, the results showed no clear evidence of genetic backcrossing toward *C. aristulifera*, reflecting the preserved species integrity of *C. aristulifera* regardless of the divergence in flower color.

### 3.6 Relationship between morphological and genetic distances

**Figure 8:**
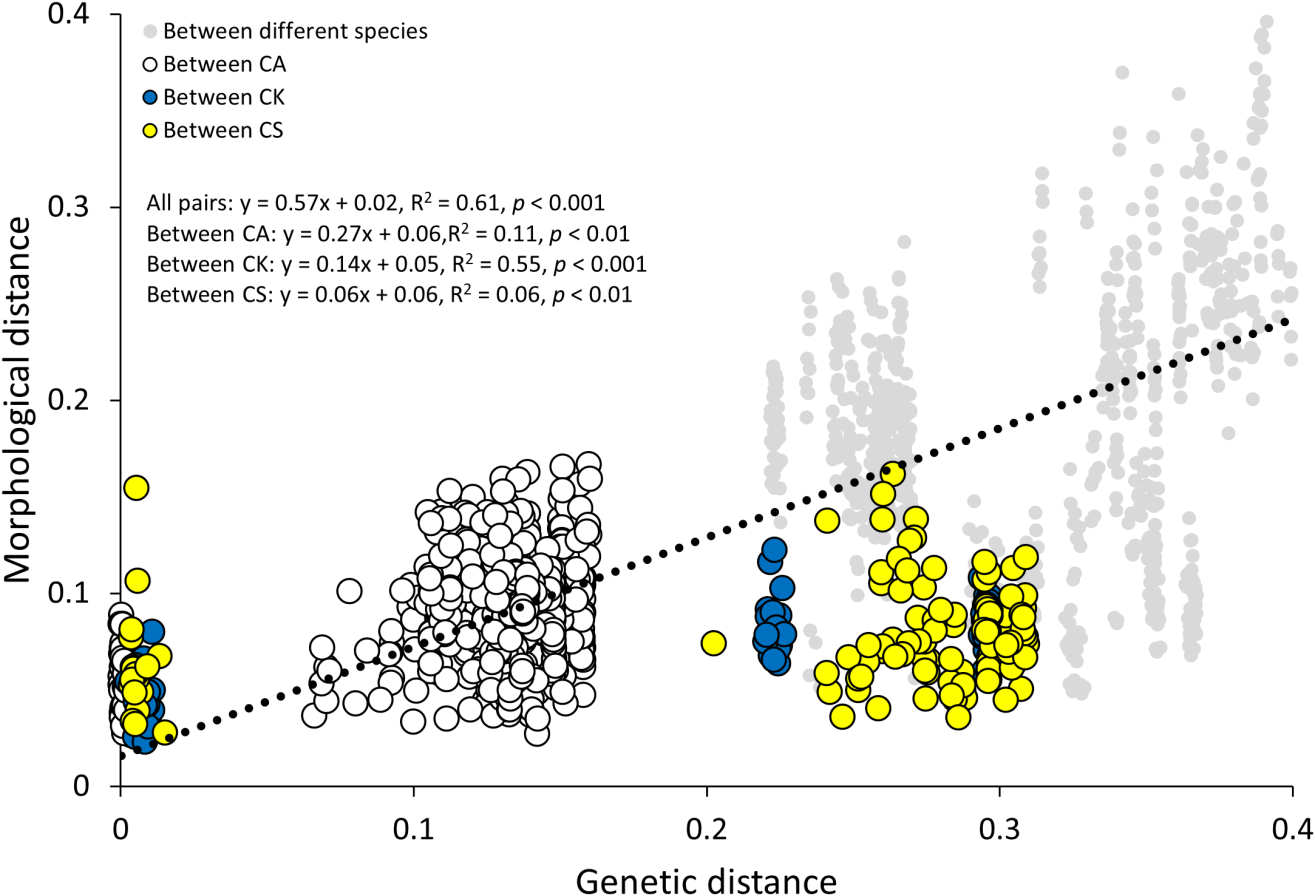
Multiple matrix regression with randomization on genetic and morphological distances based on 64 sampled individuals (CA: *Calanthe aristulifera*, CK: *Calanthe* × *kibanakirishima*, CS: *Calanthe sieboldii*). Bray-Curtis dissimilarity and Jaccard index were used for morphological and genetic distances, respectively.

MMRR indicated that floral morphological distance increased with genetic distance (R^2^ = 0.61) (**Figure 8**). Such increasing pattern was particularly distinct for the distances between *C.* × *kibanakirishima* individuals (R^2^ = 0.55) rather than those between *C. aristulifera* (R^2^ = 0.11) and *C. sieboldii* (R^2^ = 0.06) (**Figure 8**). Compared to *C.* × *kibanakirishima* and *C. sieboldii*, the genetic distance between *C. aristulifera* individuals was generally shorter (**Figure 8**). Furthermore, CAP showed that the measured floral morphological traits explained 76.6% of genetic variations across the 64 samples (**Table 2**). Independent, marginal effect analysis suggested that spur length and diameter, front and lateral lip lengths, and front and lateral lip widths were related to the genetic variability (**Table 2**).

**Table 2:** Marginal effects of floral morphological traits on the multivariate variance in 3382 SNPs in accordance with canonical analysis of principal coordinates (CAP).

Variation partitioning	Inertia	Proportion (%)
Total	1.03	100.0
Constrained	0.79	76.6
Unconstrained	0.24	23.4
Explanatory variable	F value	*p* value
Flower number	0.89	0.38
Sepal length	1.11	0.29
Sepal width	1.73	0.15
Petal length	0.71	0.49
Petal width	2.29	0.09
Spur Length	4.73	< 0.01
Spur diameter	3.04	< 0.05
Front lip length	5.59	< 0.05
Front lip width	3.88	< 0.05
Lateral lip length	7.06	< 0.005
Lateral lip width	2.92	< 0.05
Gynandrium length	1.74	0.15

## 4 Discussion

Regarding our research hypothesis, no clear difference in floral morphological traits and genetics was detected between the two phenotypes of *C. aristulifera*. The detected genetic similarity between CA1 and CA2 was similar to the preserved species integrity of *Goodyera similis* Blume. populations on an insular hybrid zone (Suetsugu et al., 2024). Furthermore, no clear differences between the two phenotypes of *C. sieboldii* coincide with PCR-based study of Srikanth et al. (2013) suggesting that a *Calanthe* mutant with yellow petal and white lips might be genetically clustered with *C. sieboldii*. These patterns show that the observed phenotypic differences between the phenotypes might not result from either historical speciation or gene exchange through natural hybridization at least for *C. aristulifera* and *C. sieboldii* (Evans et al., 2023). Instead, they might be simply related to the levels of specific gene expression associated with floral color within the studied *Calanthe* populations (Choi et al., 2019). It was also found that the genetic distance among *C. aristulifera* individuals was closer than that among *C. sieboldii* and *C.* × *kibanakirishima* individuals (**Figure 4b**, **8**). These results demonstrate that the *C. aristulifera* populations have retained their species integrity in the wild, regardless of the phenotypic divergence in flower color and size. Therefore, our null hypothesis on the relationship between the phenotypic divergence and hybridization of *C. aristulifera* is rejected.

There were, however, remarkable differences between the phenotypes of *C.* × *kibanakirishima*. Particularly, the lowered hybrid index and interspecific heterozygosity of CK2 (0.23–0.24 and 0.40–0.42) compared to CK1 (0.48–0.50 and 0.72–0.89) indicate the presence of genetic backcrossing of *C.* × *kibanakirishima* toward *C. sieboldii*. These findings are on the contrary to Oh et al. (2014, Oh et al., 2015), who speculated that *C.* × *kibanakirishima* might not be a hybrid origin according to the internal transcribed spacer (ITS) and several chloroplast DNA regions (*atp*F-*atp*H, *psb*K-*psb*I, and *trn*H-*psb*A). Instead, our results are in line with the prevailing hybridization and backcrossing among the coexisting, allied orchid species in nature (Cozzolino et al., 2006; Evans et al., 2023; Jiang et al., 2024; Johnson, 2018; Pansarin, 2025). These results support previous findings on the blurred morphological and genetic boundaries between coexisting allied orchid species due to the occurrence of backcrossed hybrid individuals in an oceanic island (Suetsugu et al., 2024).

Our results also showed that the floral morphological distance increased with the genetic distance; however, the relationship between morphological and genetic distances was remarkable for *C.* × *kibanakirishima* rather than *C. aristulifera* and *C. sieboldii* (**Figure 8**). These results indicate that phenotyping based on floral morphology could reflect the genetic variability related to the hybridization. It supports BerSweden et al. (2021), who demonstrated that the floral morphological distance between the hybrid individuals increased with genetic distance by repetitive hybridization and backcrossing in *Orchis militaris* L., *Orchis purpurea* Huds., *Orchis simia* Lam., and *Orchis anthropophora* (L.) All. hybrid zones. Meanwhile, Jiang et al. (2024) found that divergence in floral dot size and density of *Paphiopedilum wenshanense* Z.J. Liu & Yong Zhang corresponded to the genetic variation due to the interspecific introgression from *Paphiopedilum concolor* (Lindl. ex Bateman) Pfitzer. In our study, *C.* × *kibanakirishima* without reddish tint on lip (CK2) might result from the backcrossing of *C.* × *kibanakirishima* with reddish tint (CK1) and *C. sieboldii* (**Figure 1**), confirming such relationship between floral coloristic divergence and historical gene flow through interspecific hybridization. Overall, our null hypothesis on the relationship between phenotypic and genetic variations may be partially applicable for the hybrid individuals rather than the parent taxa themselves.

The observed genetic backcrossing of *C.* × *kibanakirishima* was biased toward *C. sieboldii* rather than *C. aristulifera*. This skewed pattern might result from the similarity in floral morphology between *C.* × *kibanakirishima* and *C. sieboldii*. It was found that CK1 featured the similar size of gynandrium with *C. sieboldii*, which was 38% larger than that of *C. aristulifera* (**Figure 3l**). This biased floral trait of the hybrid individuals might promote the pollen flow between *C.* × *kibanakirishima* and *C. sieboldii* more than that between *C.* × *kibanakirishima* and *C. aristulifera*, by which the interspecific hybridization could become unidirectional toward *C. sieboldii* (Minder et al., 2007). Chung et al. (2005) reported that the natural hybridization and backcrossing of the South Korean *Liparis* species was skewed toward *Liparis kumokiri* F. Maek. rather than *Liparis makinoana* Schltr. because of the similarity in floral characteristics between the F1 hybrids and *L. kumokiri*. Ellis and Johnson (1999) also suggested that difference in flower lip and spur could act as a mechanical barrier of hybridization by inhibiting pollinators from landing on the flowers and probing for potential nectar sources. We actually found that both the phenotypes of *C. aristulifera* had smaller lip and longer spur than CK1 (**Figures 3f, h, i**), and such variabilities in lip and spur sizes were the significant explanatory variables for the genetic variations (**Table 2**). These findings suggest that pollinia and gene exchange between *C. aristulifera* and *C.* × *kibanakirishima* might be relatively limited compared to those between *C. sieboldii* and *C.* × *kibanakirishima* due to the morphological dissimilarity.

It is notable that the detected hybridization in this study might be rare and restricted recently. The latest literature on our study site recorded the complete absence of fruit set and pollinia removal by insects of *C. aristulifera* populations from 2021 to 2025, due to the limitation of effective pollinators like *Eucera nipponensis* and *Lasioglossum occidens* (Kim et al., 2026). In this context, recent formation of F1 hybrid between *C. aristulifera* and *C. sieboldii* could be inactivated in the study site, indicating that the early generations of hybrids (CK1) in this study might be historically formed. Furthermore, the smaller number of CK2 (3 individuals) than CK1 (26 individuals) in the study site allow us to expect that the hybridization and backcrossing between *C. sieboldii* and *C.* × *kibanakirishima* could be occasional in the wild. These findings support Chung et al. (2005), reporting the evidences for historical hybridization and introgression between *L. kumokiri* and *L. makinoana* instead of the ongoing genetic recombination of those species. In contrast, Suetsugu et al. (2024) highlighted that the lack of effective pollinator (bumblebee) for *G. henryi* could induce inefficient and sporadic pollination by wasps, which potentially promoted interspecific hybridization with wasp-pollinated species (*G. similis*) and diversification of hybrid genotypes. Thus, long-term monitoring should be necessary to totally elucidate whether the detected genetic patterns across the studied *Calanthe* species will persist or change to more complicated hybrid swarms.

Both CA1 and CA2 featured lower genetic diversity indices than CS1 and CK1 (**Table 1**). Combining with the close genetic distance among *C. aristulifera* individuals (**Figure 4b**, **8**), the results enable us to emphasize the poor genetic diversity in the *C. aristulifera* populations despite their phenotypic divergence and species integrity. Given that this species had suffered population decline by poaching (Hong et al, 2009) and has been protected as the legal endangered species since 2017, genetic bottleneck by historically reduced population size might be one of the mechanisms left behind the poor genetic diversity (Maarten et al., 2025). This pattern is consistent with Katafuchi et al. (2024), exhibiting severe genetic diversity loss of *C. hoshii* in Ogasawara Islands, of which wild populations have been declined by illegal excavation, habitat loss, and invasion of alien species. The absence of outer genetic sources might also play a role for the lowered genetic diversity in the *C. aristulifera* populations, considering the geographic isolation of the study site as an oceanic island and little genetic introgression from the coexisting allied and hybrid species. Therefore, our results highlight the importance of maintaining genetic diversity beyond preserving species integrity for conserving wild *C. aristulifera* at the current stage.

Several conservation strategies can be implemented for enhancing genetic diversity of remaining *C. aristulifera*. Ahrens et al. (2017) recommended that managing entire gene pools beyond the species boundary of the endangered *Diuris basaltica* D.L. Jones is required to reinforce the gene flow patterns and associated evolutionary processes recovering allelic diversity. From this perspective, extensive habitat protections for *C. sieboldii* and *C.* × *kibanakirishima* can be an option to restore genetic diversity of *C. aristulifera*. *Ex situ* conservations for artificial propagation and subsequent reintroduction experiments should also be prioritized to achieve bigger wild population size and reduce the probability of geographic and genetic isolations of remaining *C. aristulifera* (Yang et al., 2014). Given the recent reproductive constraints of *C. aristulifera* in the study site (Kim et al., 2026), applying forest management practices should be considered to attract effective pollinator insects by increasing nectar plant diversity near *C. aristulifera* populations (Sakata et al., 2014). Artificial cross-breeding programs may be applicable to recuperate the poor genetic diversity of *C. aristulifera* in the short-term (López-Olvera et al., 2025).

Although our study provides valuable preliminary information regarding the relationship between floral phenotyping and genetic variability in the endangered *Calanthe* species, several limitations should be addressed by the future studies. For example, our study used unbalanced sampling design and small number of samples for several phenotypes (CK2 and CS2) because of their low occurrence in the wild (3 and 2 individuals). Artificial propagation and cross breeding experiments may be an alternative to acquire larger number of samples for the rare phenotypes and overcome such issues. In addition, our study adopted several extra clustering procedures to seek species-specific SNP groups (**Figure 6**) because of the lack of diagnostic markers for the studied *Calanthe* species. Further studies should focus on developing diagnostic molecular markers for *C. aristulifera* and allied species to simplify such additional procedures and enhance the applicability of the findings. Given that patterns in hybridization and morphological divergence can vary with the surrounding environmental gradients (Ahrens et al., 2017; Evans et al., 2023), further studies using multiple hybrid zones should be needed to confirm that the observed patterns among the studied *Calanthe* species can be detectable across the broader geographical context.

In summary, this study demonstrates that phenotyping based on floral morphology alone may not be enough to display the genetic variability and species integrity in the endangered *C. aristulifera* populations on an oceanic island. Instead, the floral phenotyping corresponded to the genetic variability in the hybrid individuals (*C.* × *kibanakirishima*), which could reflect the historical hybridization and genetic backcrossing in the wild. In particular, we find that several hybrid individuals (CK2) were morphologically closer to *C. sieboldii* than *C. aristulifera* while the opposite pattern was undetected, which coincide with the patterns in the population genetics. This indicates the occurrence of the asymmetrical hybridization and repetitive backcrossing toward *C. sieboldii* possibly resulting from the dissimilarity in floral morphology (e.g., gynandrium, lip, and spur) between the hybrid and *C. aristulifera*. In the context of conservation, our results also reflect that *C. aristulifera* featured higher species integrity and poorer genetic diversity than the coexisting allied species, regardless of the detected intraspecific variations in flower color and size. Therefore, conservation programs should focus on protecting genetic diversity of *C. aristulifera* populations from the risk of genetic bottleneck by illegal poaching and habitat loss more than preserving species integrity. The efforts for maintaining entire gene pools beyond species boundary and relieving reproductive constraints in the wild should be conservation priorities to recover genetic diversity of *C. aristulifera* in the long-term.

## Data Availability

The datasets presented in this study can be found in online repositories. The names of the repository/repositories and accession number(s) can be found below: https://www.ncbi.nlm.nih.gov/, PRJNA1306762.
